# Anticipatory governance for social-ecological resilience

**DOI:** 10.1007/s13280-014-0604-x

**Published:** 2015-01-09

**Authors:** Emily Boyd, Björn Nykvist, Sara Borgström, Izabela A. Stacewicz

**Affiliations:** 1 School of Archaeology, Geography and Environmental Science (SAGES), University of Reading, Whiteknights, Reading, UK; 2 Stockholm Environment Institute, 115 23 Stockholm, Sweden; 3 Stockholm Resilience Centre, Stockholm University, 106 91 Stockholm, Sweden

**Keywords:** Anthropocene, Anticipation, Governance, Climate change, Knowledge, Networks

## Abstract

Anticipation is increasingly central to urgent contemporary debates, from climate change to the global economic crisis. Anticipatory practices are coming to the forefront of political, organizational, and citizens’ society. Research into anticipation, however, has not kept pace with public demand for insights into anticipatory practices, their risks and uses. Where research exists, it is deeply fragmented. This paper seeks to identify how anticipation is defined and understood in the literature and to explore the role of anticipatory practice to address individual, social, and global challenges. We use a resilience lens to examine these questions. We illustrate how varying forms of anticipatory governance are enhanced by multi-scale regional networks and technologies and by the agency of individuals, drawing from an empirical case study on regional water governance of Mälaren, Sweden. Finally, we discuss how an anticipatory approach can inform adaptive institutions, decision making, strategy formation, and societal resilience.

## Introduction

Anticipation has been widely studied within numerous different fields, and under diverse names, in fields including biology, psychology (Louie 2009; Louie and Poli 2011; Poli 2009, 2010, 2011), resilience (Almedom et al. 2007; Almedom 2009; Martin-Breen and Anderies 2011; Zolli and Healy 2012), Future Studies (Miller 2006, 2007, 2011, 2012), and governance (Fuerth 2009, 2011; Karinen and Guston 2010; Fuerth and Faber 2012).

All attempts to understand, imagine, and benefit from the future can be seen as modes of anticipation, a constant feature of human behavior (Poli 2011). Prophecies and ideas of imaginable futures are the focus of substantial current discussion, e.g., ‘forecasting’ financial markets, or modeling Earth’s ecological boundaries. Such anticipatory practice, in situations of noteworthy and alarming change, are conceivably highly beneficial to imagine how to elucidate complexity and decipher ‘wicked’ problems, and engage with new mechanisms to harness the future. Early exploration of anticipatory practice suggests that anticipation potentially helps to raise awareness about the types of futures mankind may encounter and sensitize society to the consequences of choices and actions of individuals and societies (Poli 2009, 2010, 2011).

To date, there have been partial systematic efforts to construct an in-depth understanding of different forms of anticipation, their uses and risks. The research foundation is in progress, but it is disjointed (Poli 2010). In the cognitive sciences, Gilbert and Wilson (2007) have proposed the controversial notion of ‘prospection’—the psychology of imagining the consequences of hedonic future events (Fukukura et al. 2013). Critics of Prospection Theory say it reflects deterministic explanations of cognition, as it does not advance conscious decision making or agency. The field of Futures Studies focuses on building a theory of adaptation where we still lack understanding about how societies cope, prepare, and adapt to change (Floyd 2012). This field is generally understood to be strong on practice and facilitation of scenarios rather than on its theoretical foundations. The field of social-ecological resilience believes that humanity is now influencing every aspect of the Earth on a grand scale (Rockström et al. 2009), which is aligned with many broader fields, including geography (Goudie 1989, 2013; Turner 1990), biological and environmental sciences (Vitousek et al. 1997), and economics (Swanson 1996). The planet has entered a new geological era called the Anthropocene (Zalasiewicz et al. 2010). Human impacts on the planet are thought to be significant, interconnected in complex ways, containing a risk of an irreversible and uncertain sequence of changes, leading societies into a profoundly different future to anything experienced by humans in the past (www.anthropocene.info/en/home). Berkes et al. (2003) say “The challenge is to anticipate change and shape it for sustainability in a manner that does not lead to loss of future options” (p. 354). Hence, anticipation is a critical component for building resilience. Yet, apart from a few exceptions (Tschakert and Dietrich 2010), resilience literature does not drill down into theories and approaches that explore the relationship between anticipation and adaptation, in decision making and planning for environmental futures.

This paper seeks to identify how anticipation is defined and understood in the literature and to explore the role of anticipatory practice to address individual, social, and global challenges. In particular, we focus on the importance of anticipation to building resilience of coupled ecosystems and livelihoods under a changing climate by developing an approach that is capable of framing and enhancing the potential of anticipatory practices. Our work is primarily contextualized within the social–ecological systems (SES) research field, but our review of anticipation is broad and the empirical case focuses on the aspects of anticipation of generic interest, also for policy science, planning, and futures studies. The study’s overarching goal is to contribute to wider discussions of defining and studying anticipation empirically.

Our research questions used to explore these aims are as follows: Firstly, in theory, how is anticipation defined and understood, and to what extent is anticipation considered a core mechanism for adaptation in SES? Secondly, in practice, how anticipatory are governance structures? i.e., how do organizations and government agencies anticipate changes to vulnerable ecosystem services (e.g., water) in the Mälardalen Region of Stockholm, Sweden, and adapt governance accordingly?

The paper is set out as follows. The next section defines anticipation and describes literature on anticipation and SES resilience and the relationship with existing forms of governance. The case study is then presented, with findings from the Mälardalen region in Sweden. The paper discusses these results and speculates on the risks and uses of an anticipatory approach. The paper concludes with thoughts on the potential opportunities to lay the foundation for understanding anticipation to enhance decision and policy making for uncertain futures.

## Materials and methods

Firstly, we conducted an extensive review of the literature by searching for key words ‘anticipation’, ‘adaptation’, ‘resilience’, ‘climate governance’ and a combination of these on the Web of Science and Google Scholar broadly across areas of anthropology, biology, psychology, philosophy, and physics, and specifically on SES resilience, governance, planning, and futures to identify definitions of ‘anticipation’ and criteria to examine anticipation as an approach. We selected the case study of water governance and early warning network configurations in ecosystem management in the Mälardalen region, Sweden. We studied the actors and institutions in the urbanizing Mälardalen region and their capacity to govern resources under uncertain change. This provides a fascinating case of potential anticipatory practices for individuals, organizations, and society. We studied the actors involved in governing water and those who use water-related ecosystem services at the regional scale. We conducted qualitative interviews (*n* = 21 including two Stockholm region municipalities) over 10 months during 2013. Interviews were analyzed with open coding using the ATLAS.ti software to identify key patterns (Coffey and Atkinson 1996; Patton 2002) on anticipatory behavior, foresight, and adaptation to novel changes. Criteria selection for our case included: geographical scale (regional drainage basin), governance scale (multilevel governance system, focus on regional actors), water quality (engaged in governance of), actors (influencing or engaged), and the system being affected by climate change. Given these boundary conditions, the study aimed to interview the complete set of relevant regional actors around Lake Mälaren in Mälardalen region. Limitations of the study include the possibility that all relevant actors were not identified through our scoping and review work and through the snowball method used (Noy 2008). We were also resource constrained in the number of interviews possible with larger actors, e.g., County Administrative Board, which fulfills different functions.

## Results

### Defining ‘Anticipation’

Anticipation has been widely studied within numerous different fields and has been described as a discipline in its own right (Miller et al. 2013) (Table 1). While rooted in theoretical biology, Rosen’s (1985) Theory of Anticipatory Systems appears across fields, and has been extensively applied to human systems. While acknowledging that little is understood about anticipation, Poli (2010) shares the following conclusions:

**Table 1:** Definitions and approaches to anticipation

Fields	Definition	Themes addressed	Sources
Philosophy	According to Husserl, anticipation is the way in which the merely co-presented is present in perceptual experience. Heidegger’s “Philosophy of Death” describes anticipation as “the possibility of understanding one’s own most and uttermost potentiality-for-Being-that is to say, the possibility of authentic existence”	Anticipation as a component of consciousness; humans’ expectations	Husserl (1991), Bloch (1995), Heidegger (1962, p. 260)
Biology	Rosen’s Theory of Anticipatory Systems states that: “An anticipatory system is a system containing a predictive model of itself and/or its environment, which allows it to change state at an instant in accord with the model’s predictions pertaining to a later instant.” His theory showed that anticipation is not limited to living systems. Poli (2010, p. 8) states, “non-living or non-biological systems can be anticipatory”	Theory of Anticipatory Systems	Rosen (1985, p. 341), Louie (2009), Louie and Poli (2011), Poli (2009, 2010, 2011)
Psychology	The psychology of imagining the consequences of hedonic future events and future orientation of cognitive studies	Cognitive studies	Fukukura et al. (2013)
Physics	Dubois (2000) distinguishes between weak anticipation: when systems use a model of themselves for computing future states; and strong anticipation: when the system uses itself for the construction of its future states. With strong anticipation, anticipation is no longer similar to prediction (see planning below)	Anticipation can stabilize otherwise unstable states; Anticipation is stored in a system’s potential energy	Dubois (2000), Ferret (2010)
Anthropology	In relation to climate change, Nuttall (2010, p. 23) states, “While adaptation is largely about responses to climate change, anticipation is about intentionality, action, agency, imagination, possibility, and choice; but it is also about being doubtful, unsure, uncertain, fearful, and apprehensive.” Nuttall finds that anticipation may be a prerequisite for thinking about CCA	Anticipation to orient human action; how people make choices and decisions based on predictions, expectations or beliefs about the future	Bennett (1976), Nuttall (2010)
Resilience	Anticipatory adaptation acts on the best models of climate change impacts. They “are effective in creating systems that are able to maintain their state in response to the unexpected crises arising from climate change” (Martin-Breen and Anderies 2011, p. 48)	Anticipation is an important feature of resilience. Resilience literature mentions anticipation but does not seem to draw extensively upon anticipation theory	Almedom et al. (2007), Martin-Breen and Anderies (2011), Berkes et al. (2003)
Futures, planning	According to Fuerth (2009, p. 29), anticipatory governance is “a system of institutions, rules and norms that provide a way to use foresight for the purpose of reducing risk, and to increase capacity to respond to events at early rather than later stages of their development”	Anticipatory governance; forecasting, simulation, trend extrapolation, scenarios. Anticipation is well developed in this field	Quay (2010), Fuerth (2009), Karinen and Guston (2010), Miller (2006, 2007, 2011, 2012)

1. Anticipation comes in different forms, e.g., explicit and implicit, and different types of anticipation may work simultaneously.
2. Anticipation has been a major evolutionary breakthrough. If Rosen’s theory (1985) holds true, anticipation may be deeply embedded in the organisms’ functional structure.
3. Anticipation’s abstract nature depends on hierarchical, or self-referential loops, imposing severe constraints on the modeling of anticipation systems.

Rossel (2010) stresses that the anticipatory systems concept is another way of framing reality, so even with highly sophisticated modeling tools, we cannot escape our inability to be outside ourselves.

#### Anticipation and resilience in Social-Ecological Systems

The IPCC (2012) defines resilience as “the ability of a system and its component parts to anticipate, absorb, accommodate, or recover from the effects of a hazardous event in a timely and efficient manner.” Broadly, literature points toward SES resilience encompassing anticipation, e.g., in an examination of the effects of climate change in Africa; Conway (2008) argues that building resilience starts with anticipation, surveying, and forecasting (as has long been used in addressing natural disasters). Anticipation was mentioned, but not elaborated on, as a key challenge in the seminal book Navigating Social–Ecological Systems (Berkes et al. 2003). Rogers (2011) highlights how anticipation and assessment, alongside preparation and prevention, are key features of pre-emergency event aspects of resilience. Rogers defines anticipation as “horizon scanning to identify potential dangers, registering those in a formal typology and recognition of the changing nature of risks that need to be continually identified and re-assessed”. Rogers argues that hazard anticipation should be included, with risk assessment, into the strategic framework for emergency management. Tschakert and Dietrich (2010) describe anticipatory learning, which falls under the umbrella of ‘action learning’, as a crucial element for climate resilience. However, they argue that resilience thinking and anticipatory learning have occurred in parallel rather than in synergistic ways and could be more effectively integrated.

There is some consistency in the definition of anticipation in the context of resilience, but definitions vary between anticipation meaning foresight, preparedness, and planning practices (Wardekker et al. 2010), and being predictive/proactive, in contrast to adaptation (Nuttall 2010). Nuttall (2010) describes anticipation as being about foresight, rather than expectation, as anticipation draws upon predictive capabilities, knowledge, experience, and skill. Anticipation is described as being about “intentionality, action, agency, imagination, possibility, and choice; but it is also about being doubtful, unsure, uncertain, fearful, and apprehensive.” This literature distinguishes between foresight and prediction, with foresight emerging as an important strategy in building adaptive capacity. As Wardekker et al. (2010) note, “planning and foresight/research are important instruments of anticipatory adaptation, which is specific to human rather than natural systems,” and other scholars such as Hill (2013) have similar distinctions. In building resilience, Wardekker et al. (2010) emphasize the importance of the flow of foresight information/research both from and to local practitioners.

Gómez-Baggethun et al. (2012) explain that according to resilience theory, traditional ecological knowledge (defined as, “the body of knowledge, beliefs, traditions, practices, institutions, and worldviews developed and sustained by indigenous, peasant, and local communities in interaction with their biophysical environment”) evolves over time, on the basis of long-term observation and responses to crises. Long-term observation can therefore feed into traditional knowledge, necessary for resilience, suggesting links between traditional knowledge, anticipation, and resilience. However, little literature makes these linkages in a climate resilience context. An understanding of ecological knowledge seems to only be implicitly stated in current resilience literature on climate futures. Nuttall (2010) also notes that little of the anthropology of anticipation appears to have entered climate change discussions. Using the example of a mine spill of the Aznalcollar tailings dam, Gómez-Baggethun et al. (2012) state that in order to deal effectively with increased uncertainty due to environmental change, new governance approaches should use traditional ecological knowledge and utilize the social–ecological memories (the accumulated experience of knowledge and institutions) of local cultures. This memory complements current science and technology in creating governance systems relevant to local contexts, contributing to long-term social–ecological resilience. Unlike much of the extant resilience literature, Gómez-Baggethun et al. attempt to tease out what needs to be done to build resilient governance structures.

Wyckhuys and O’Neil (2010) emphasize the importance of combining farmers’ and scientists’ ecological knowledge in mutual learning systems, to better understand the workings of local agroecosystems. While the relationship between anticipation and traditional ecological knowledge is broadly missing from resilience literature, Valdivia et al. (2010) make the more explicit linkage between traditional knowledge with anticipation, implying that new traditional local knowledge informs adaptive processes. This requires an assessment of traditional knowledge, development of future scenarios, and use of participatory research to identify alternative adaptation strategies.

The literature clearly indicates that anticipation is a critical component for building resilience. The use of local ecological knowledge in the design of governance frameworks for climate resilience is important. Anticipation systems may be more effective if an understanding of local ecological knowledge is considered. Folke et al. (2005) describe networks and social learning in a less-defined way, whereas futures studies and some other resilience scholars are more prescriptive.

#### Anticipation and governance

Over the past decade, resilience scholars have focused on the concept of adaptive governance when studying how societies interact with and govern ecosystems (Folke et al. 2005). Adaptive governance encompasses and identifies adaptive response strategies associated with uncertain environmental risk, and an important feature is that societies are flexible in their responses to environmental crises. Governance includes “all processes of governing, whether undertaken by a government, market or network, whether over a family, tribe, formal or informal organization or territory and whether through laws, norms, power or language” (Bevir 2013). Adaptive governance requires that governing processes take place through nested and networked governance structures. Polycentricity contrasts with traditional top-down approaches and requires the creation and dissemination of detailed and current bottom-up information to support central decision-making processes (Ostrom 2010). This is evident in the emergence of ‘citizens as sensors’ (Goodchild 2007). Citizen science describes bottom-up communities/networks of citizens acting as observers in some scientific domain. For instance, in the US, many farmers now have more elaborate, detailed, and current mapping and monitoring systems for their fields and crops than those held by central agencies. In a successful climate, early warning system in the Sahel, a bridging organization facilitated a network of government, scientists, NGOs to provision and process real-time monitoring (RTM) rainfall data relevant to communities, with those who could take preemptive early action to build resilience in the face of recurring crisis (Boyd et al. 2013). Understanding adaptive governance has helped different communities to better coordinate practices of living with uncertain futures.

More recently, there has been a policy shift toward understanding climate adaptation and uncertainty in the context of forecasting/predicting change. This is aligned with growing knowledge about attributing impacts of extreme events to greenhouse gas emissions (Stott et al. 2013). As someone must bear the costs of the consequences of climate change, it becomes more imperative to forecast or anticipate the future based on current knowledge, models, and creative imagination. Methods and approaches for better anticipating future changes are in demand. Tschakert and Dietrich (2010) state that “identifying and monitoring slowly changing variables such as rainfall patterns and integrating and reflecting on new knowledge allows for a better understanding of processes that are already underway. The same is true for anticipating possible events assuming observed trends continue. Monitoring enhances flexibility during times of disturbance and boosts the capacity for anticipatory action.” In alternative Futures Studies for the healthcare sector, Bezold and Rowling (2008) find that biomonitoring devices could play a large role in achieving disparity reduction across income and racial/ethnic lines in the US.

Anticipatory governance is a new concept that has significant relevance for developing strategies under uncertain environmental futures. Anticipatory governance involves changing short-term decision making to a longer-term policy vision, including the notion of foresight. Quay (2010) states that a wide range of futures is anticipated in anticipatory governance—assessment/analysis is undertaken across a range of scenarios (using criteria of aggregation, extremes, sensitivity, risk assessment). Multiple strategies are anticipated, which are appropriate in the short and long term, given the range of possible futures. Changing conditions are monitored over time. Key precursors are identified, associated with various possible futures. It is important for managing events instead of waiting until a climate-related or regulatory/socio-economic event results in crisis. For example, the health sector has shown that coupling anticipatory governance with RTM can ground anticipatory outlooks in important ways. This involves “co-production” of knowledge, jointly designed by experts and citizens linking the evidence base or informed decision making to management. While the concept of anticipatory governance is important, it is also important to calibrate predictions. In the context of resilience and governing ecosystem services (e.g., water) under climate change, such a framework has yet to be articulated.

Anticipatory governance also features in Futures Studies, which includes all the ways to study, think, and use the future—ranging from visionary and utopian futures to pop futures, from participatory, critical, or integral futures to the technicalities of simulations, formal modeling, and forecasting. Future Studies is inclusive. Every aspect, type, and way of including the future within one’s analysis, theories, or actions is a legitimate component of this field. However, some components of Future Studies are more subject to constraints than others. In particular, exercises conducted by professional futurists and the formalized transmission of existing knowledge through teaching require forms of accountability that need not constrain the field as a whole—such as responsibility toward clients and students, and basic research. Aspects of Future Studies address the human use of anticipation, either as an applied activity or as a learning process in the context of the environment.

### Anticipation through regional water networks

Next, we set out the common parameters for assessing anticipatory governance through an empirical case study on regional water governance of Mälaren, Sweden. The case study explores how anticipatory are governance structures? In other words, how do organizations and government agencies anticipate changes to vulnerable ecosystem services (e.g., water) in the Mälardalen Region of Stockholm, Sweden, and adapt governance accordingly?

#### Case background, ecosystem services, and regional Climate Change Adaptation (CCA) in Sweden

In Sweden, CCA has gradually become more important. Before 2008, no agencies had a mandate to work on CCA (Simonsson et al. 2011). The turning point was the instrumental Commission on Climate and Vulnerability (SOU 2007, p. 60) which highlighted the increased AQrisk of, e.g., flooding, and thus growing challenges for the region’s physical infrastructure and drinking water provisioning (RUFS 2010). It also recognized ecosystem challenges (Simonsson et al. 2011). In 2008, County Administrative Board was tasked with coordinating CCA (Government Bill 2008/2009, p. 162; Simonsson et al. 2011; André et al. 2012). Mälaren supplies drinking water to 2 million people (about a 1/5th of the Swedish population), but the region and its water ecosystem services are threatened by climate change. Importantly, in Sweden, there is no national authority with overarching responsibility, so CCA is primarily a regional level governance issue.

#### Actor networks, formal governance networks in water management

**Fig. 1:**
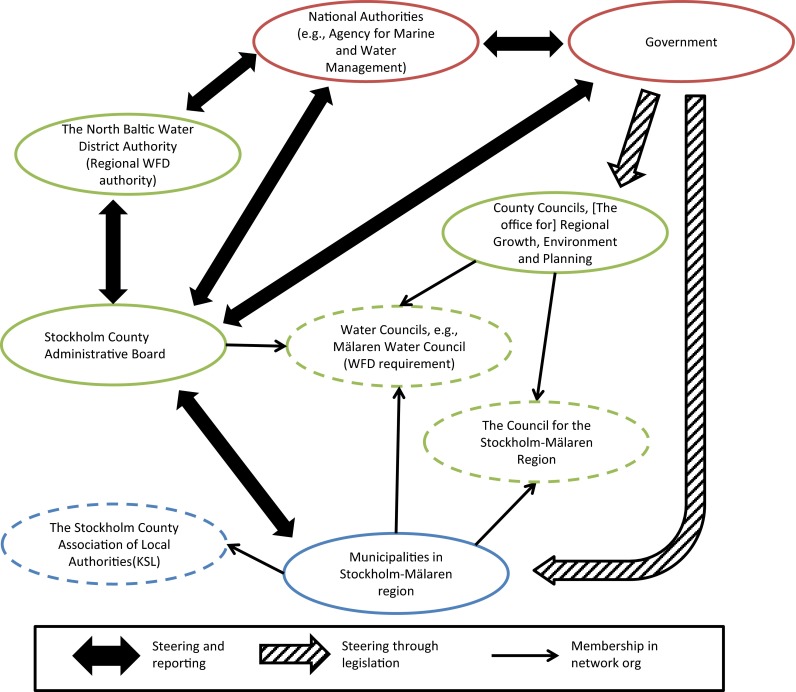
The formal governance networks in Stockholm region (adapted from Nykvist et al., unpublished results)

Most of the relevant formal actors for CCA and management of ecosystem services are part of established governance structures, rather than being a response to changing futures and unknowns. The County Administrative Board is the most central actor with a mandate to coordinate CCA (Fig. 1). However, the municipalities and local water councils and collaborations between these actors, when water crosses jurisdictional boundaries, are the most important actors for water management, and for realizing CCA. They have autonomy and planning responsibility, and a local understanding of problems and the municipalities. The Swedish setup is fairly tuned with theory highlighting the importance of government and central nodes in the network for overview and strategy, but smaller and more localized nodes are more important for generating timely and detailed understanding of the system. However, the question is how anticipatory are these governance structures in practice?

#### Type of anticipatory practices identified

Three levels of anticipatory governance are recognized, from a minimal form, representing the constant gradual adaption to immediately foreseeable changes in discourses, to the most proactive, flexible, and open strategies.

*Constant adaptation (incremental change)* is the most grounded answer in the interview data across all actors when probing for explanations of dealing with uncertainty. “The processes that generate new knowledge about drinking water and ground water are in constant change” (Informant 12^1^). Anticipation can, in this case, only be regarded as happening within the current framing of problems. In turn, the problem framings change gradually. Anticipation is therefore close to organizational change in the form of single-loop learning (Argyris and Schön 1978) defined as learning that “refers to an instrumental change in strategy within the constraints given by overall norms and beliefs” (Pahl-Wostl et al. 2007). When crisis unfolds, adaptation can be more rapid: “With an extreme event, work [procedures] changes, e.g., flooding” (Informant 7, Stockholm County Board Administration). However, anticipation is limited, and flexibility is not necessarily pre-existing, but evoked when crisis strikes. Common examples given by actors interviewed at the regional level highlight the response structures and functions in place to manage urgent crises, such as larger accidents and weather events. “The high [water] levels of the winter 2000 that threatened the subway in Stockholm. It resulted in faster planning of renovating Slussen [the lock between Mälaren and the Baltic Sea in central Stockholm]. But this is a crisis situation, and the Emergency Response manages that together with the County Administrative Board. It is a different organization in the event of a crisis, not just planning” (Informant 1, Mälaren Water Council).

The *forecasts and projections of environmental change* may depend on issues being on the political agenda, and in the data, a strong theme is that global CCA is not prioritized. A focus on global and national CCA in general, and the challenges for ecosystems, and regional and local water ecosystem services, is clearly lacking. “We do not work explicitly with CCA. It is due to the [lack of] interests among the municipalities” (Informant 6). Anticipation, in terms of the forecast and scenarios developed across the formal actors in the regional network, are heavily focused on established political development priorities. The focus is currently on challenges for the built environment, such as flooding of infrastructure and saltwater intrusion from the Baltic Sea, rather than wider, global CCA. The authorities’ scenarios of regional development drive analysis of issues of key importance, e.g., water as drinking water. The clear difference in political support is expressed by Informant 19 at the Swedish Agency for Marine and Water Management National authority: “Climate adaptation has two sides. What is most talked about is to protect people and infrastructure, but climate adaptation for ecosystems is very seldom talked about.”

Futures being produced, such as long-term planning with room for complexity are found in four key areas of strategy development. Most futures discussed relate to near-term policy objectives and targets, but longer time horizons are used in planning. They span a range of spatial scales, from EU to the Stockholm region and Lake Mälaren:

1. EU level: Water Framework Directive steers planning horizon and anticipation of problems on this time scale (2021, 2027)
2. Swedish government and steering of agencies: Polices and policy discourses as put forward in legislation and bills. Major thematic bills are reviewed every 5–10 years, but CCA and Ecosystem Services are recent to rise to the agenda and have so far only had one iteration:
  - Example 1: Recent ecosystem services bill Government Bill (2013/2014) is the first of its kind.
  - Example 2: CCA Government Bill (2008/2009)
3. County Councils and Regional planning office (Regionplanekontoret 2010): 5-year plans, and long-term scenarios, regularly updated and with some aspect of CCA included due to physical adaptation.
4. County Administrative Board: Work with long-term scenarios on climate change and flooding with CCA clearly included due to physical adaptation.

*Proactive learning, new ideas, and strategies* through networking is very important, the flexibility of institutions (Folke et al. 2005; Boyd and Folke 2012) is high, but it is demanding for the central actors coordinating these efforts. We identify a wide range of networks and collaborations, between municipalities and counties that constitute multilevel governance structures (Nykvist et al., unpubl. results). This enables a high degree of stakeholder involvement and open forms of governance to ensure learning from others’ experiences. The regional scale and its many collaborations offer platforms for spreading knowledge. The drawback is a clearly expressed lack of coordination, “There is a need for all actors to present the same message regionally” (Informant 12) and expectations of direction from national agencies. Having no national actors with overall responsibly of CCA constitutes an unresolved challenge. “The role of HAV [Swedish Agency for Marine and Water Management National authority] in relation to the regional water authorities is currently very unclear, HAV is supposed to provide steering and coordinate at the national level” (Informant 5).

*Learning/the way people are learning:* Interactions lead to awareness of complexity, but learning as in feedback from other stakeholders and feedback from past changes (physical and organizational) is limited. There is evidence that knowledge generated is not fed forward to the next iteration of problem solving and learning. Since the problems are wicked and complex in character, and individual actors do not possess all the knowledge needed, feedback is necessary. Overall, the lack of feedback through time is one of the most problematic issues and a highly grounded theme in the case data. “We do have monitoring programs. […]. But these should be increased and strengthened. […] Actually, one should have a cycle. Plan, take actions, and then follow up” (Informant 8, Västerås County Administrative Board). This lack of feedback limits how knowledge can build and transmit social-ecological memory over time (Barthel et al. 2010).

#### Risks and trade-offs (barriers)

Throughout our interviews, complexity is seen as a barrier, which limits the most anticipatory forms of governance in Table 2. There is a strong demand for reductionist approaches, reducing the level of complexity, delivering knowledge in a simpler, more accessible format: “The whole picture is not grasped. You don’t have the time. It is too big. You have a given specialty, but you can’t keep track of the whole picture” (Informant 7, Stockholm County Board Administration). Almost every actor interviewed has their own media (newsletters, magazines, policy briefs, report series, etc.) to summarize and disseminate knowledge. The demand for accessible knowledge is expressed as necessary to influence policy making, and is found in other studies of the Swedish science policy interface (Nykvist and Nilsson 2009). As the problems are complex, and therefore seldom reducible, this acts as a barrier. A concrete example is the commonly expressed view of ecosystem services as a new concept that aims to clarify the link between natural resources and our use of them. It is seen as too academic, uncertain, and not yet useful, and its use is not widespread. Trade-offs between different societal priorities, or between different ecosystem services, are therefore not illuminated as intended with the ecosystem services framework.

**Table 2:** Anticipation identified among individuals, actors, and organization

Actor	“Minimal AG”—constant, reactive, adaptation	“Some AG”—forecasts/visions	“More AG”—proactive open learning
Government and national agencies	Slow, continuous adaptation to changing discourses, e.g., CCA gradually put on the agenda	Forward-looking analysis in SOUs, but many years between major revisions and projections. In-flexible structures	Highly institutionalized procedures of peer review of polices and participation. Timeframe of mandate periods and elections severe barrier. Lack of political mandate clear barrier to fundamental learning
County Council, Regional Planning office	Crisis drives adaptation, governance is demand driven	Well developed mid- to long-term scenarios, but scope limited by current political priorities, and CCA not included	Highly participatory and collaborative processes
County Administrative Board	Crisis drives adaptation, governance is demand driven	Well developed mid- to long-term scenarios, but scope limited by current political priorities, and CCA dominated by “known” and recognized problems for physical planning	Highly participatory and collaborative processes. Quite some feedback processes, and is the key actor for facilitating learning. Dialog and arenas for learning common. Some challenges with coordination of overlapping regional processes and complexity are barriers

The most important barrier linked to futures and visions in Table 2 is that of real politics. CCA is on the agenda among some actors and in some regard. In a wider sense, and in relation to challenges to ecosystem services, CCA is much less developed. Since these complex problems require coordination, anticipatory governance developing vision hinges on a strong enough mandate being given to actors to coordinate. This is currently lacking at both the national and regional levels for CCA in a wide sense. The mandate of the County Administrative Board is clearly focused on CCA for physical infrastructure.

## Discussion

There are varied and conflicting understandings of anticipating, predicting, and forecasting futures. We discuss how anticipation can potentially improve our understanding of living with uncertain futures and where gaps lie.

### What have we learned?

This paper sets out to examine two questions. Firstly, in theory, how is anticipation defined and understood, and to what extent is anticipation considered a core mechanism for adaptation in SES? Secondly, in practice how anticipatory are governance structures? i.e., how do organizations and government agencies anticipate changes to vulnerable ecosystem services and adapt governance accordingly?

#### Lessons from the literature

We explored that an anticipatory approach is potentially helpful for improving our foresight capacity and in the co-design of solutions relevant to managing ecosystem services under climate change. The analysis mapped out different forms of anticipation from the literature and identified varied and conflicting understandings of predicting and forecasting futures. Definitions of anticipation vary and a unified definition does not exist (Poli 2010). In the relationship between anticipation and resilience, many of the literatures mention anticipation, but authors provide limited detail about how to build resilience using anticipatory systems/theory of anticipation. For example, Almedom (2009) define resilience as “the capacity of individuals, families, communities, and institutions to anticipate, withstand and/or judiciously engage with catastrophic events and/or experiences; actively making meaning out of adversity, with the goal of maintaining ‘normal’ function without fundamental loss of identity.” Anticipation plays a key role in this resilience research, but is treated in a superficial manner.

The review helped us to clarify how anticipation is both an active sense-making force and a way to anticipate dimensions of the present, with potentially important implications for the decision-making and choice-related questions at the heart of collective action (and inaction). It is imperative to continue unpacking the theory of anticipation with regard to how it features as a core of everyday social relations, affects the ability to plan under uncertainty, and contributes to adaptiveness (Folke et al. 2005; Boyd and Folke 2012). There is further scope to elaborate on a theory of anticipation and how it relates to social-ecological resilience. The review unearthed significant attention to the role of social–ecological memory, local knowledge, and anticipation. For example, Gómez-Baggethun et al. (2012) say that new environmental governance approaches should use traditional knowledge and social–ecological memories of local cultures. Linking research on social–ecological memory and anticipatory governance would benefit from further focus.

Many fields are looking at anticipatory governance, including public health (Ozdemir et al. 2009), geography (Goodchild 2007), biodiversity conservation (Barlow et al. 2010), and climate change (Boyd and Cornforth 2013). Themes are emerging around citizen science, networks, and volunteering of data sharing. In many parts of the world, networks act as local early warning systems in the face of a changing environment, ranging from disease detection, e.g., Ash (*Fraxinus excelsior*) dieback, and RTM to help governments detect early onset of famine (Boyd et al. 2013). To avoid a narrow framing of anticipation, it will be important to draw insights from Futures Studies to develop further explanations, with relative clarity, of anticipation, and anticipatory governance. Borrowing methods and tools from Futures Studies, we hope to better understand critical relationships, e.g., between the role of anticipatory governance and agency in building adaptive capacity. Future Studies is generally considered to be strong on practice and facilitation rather than on theoretical foundations. Thus, we can also draw on the rapidly emerging field of ‘sustainability transitions’ (McGrail 2012) informed by complex systems and governance theory (Loorbach 2010). Sustainability transition adopts a long-term perspective for short-term development (i.e., developing long-term visions and backcasting from them) and focuses on alternative ‘images of sustainability’ and associated possible ‘transitions paths’, and seeks to mobilize actors and instigate associated experiments.

#### Lessons from the case study

Our case of Lake Mälaren explored the anticipatory elements used by formal actors in a developed country pursuing initial work to adapt governance to anticipated future climate change. The case study shows that managing complexity for problems where anticipatory governance is needed, such as climate change, is inherently difficult; it requires both the openness and participation of adaptive governance, and coordination and simplification of knowledge to be able to make credible predictions and create and share future visions/scenarios. There is inbuilt tension between the need for complexity and the requirement for such anticipatory elements being comprehensible and easily accessible. Problem awareness is often high, and the lack of priority given to CCA and the challenges for ecosystem services is more due to lack of capacity to imagine and comprehend complex futures than out of ignorance. A longitudinal temporal framework matters to improve governance actions to respond to CCA combined with RTM of environmental events (through networks).

Collective and complex collaboration in anticipatory forms of governance puts even more requirement on coordination. Stakeholder integration with active participation and adaptive forms of governance is an increasingly common approach and observed in our case, but knowledge and learning are limited by the lack of feedback over time. As concluded by others, there is a great risk that social learning approaches does not give lasting effects (Nilsson et al. 2012). Long-term time horizons are found in the anticipatory elements of our case, and we believe that time scale per se is not the problem. Policy makers are well aware of the challenge with analyzing problems across space and time and the uncertainties this introduces. The challenge lies at the limited capacity to develop a wider range of discourses and policy problems simultaneously.

We are left with a profoundly political question of “how are the overall goals of government or society chosen in the first place?” (Toffler 1970). What will it take to move toward an anticipatory approach in which agency of individuals is connected into systems of governance genuinely producing effective social-ecological outcomes? Ultimately, the challenge is to reconcile the ‘enclaves’ of the past and future—i.e., overcome societal resistance to change and find mechanisms for societies to break away from unsustainable traditions, and learn and build decision support that engages with uncertain futures. This may require “a revolution in the very way we formulate our social goals” (Toffler 1970).

### Risks and limitations of an anticipatory approach

We identify a number of limitations to the anticipatory approach. Firstly, the literature review reveals divergent views on what anticipation means and ambiguity of meaning. This relates to the absence of theory and lack of empirical cases of anticipatory approaches to date. Secondly, anticipatory approaches in the context of resilience could be criticized for being deterministic (overlooking agency) or predetermined in that people cannot question sustainability as the end goal. This reflects criticism encountered in the lack of attention to agency in resilience literature. Critics argue that both the multiple scales of system complexity and human agency (individual and collective) need to be more thoroughly explored if resilience is to continue to have resonance more broadly (Jerneck and Olsson 2008; Hornborg 2009; Davidson 2010).

There are also limitations to ‘the practice of anticipation’, which require further exploration. In the case study, complexity is seen as a barrier, which limits the most anticipatory forms of governance. Through an SES resilience lens, we identified a strong demand for knowledge in a simpler, more accessible format. The most significant barrier is that of real politics. CCA is on the agenda, but not in a wider sense; in relation to challenges for ecosystem services, anticipatory analysis is lagging behind. Anticipatory governance developing vision requires a strong enough mandate in order for actors to coordinate. This could be one of the most challenging components to building anticipatory governance, as many actors are willing to work on the issues, but there are few incentives for sharing and building toward a common vision. Looking forward, we seek to explore ways to avoid the risks and limitations of anticipation and enhance future understanding. This challenge could be facilitated with the assistance of complementary approaches touched on in this paper, including well-established theoretical approaches and new futures methods and anticipatory actions.

## Conclusion

Anticipation has been widely studied within a number of different fields and the research base is in development, but it is fragmented. This research explored the importance of anticipation in the literature and in an empirical case study from Sweden. Anticipation is defined in different ways depending on the field. Social–ecological memory features strongly in the SES resilience literature. There is scope for further development of anticipatory theory. In practice, there is evidence of anticipatory governance operating within existing structures, yet there are limitations, such as a desire to reduce complexity, lack of effective coordination mechanisms, and real politics. Further development of tools and methods are required from across a range of fields to overcome these limits, and to lend insights about how to do this in ways that address politics, complexity, and individual and collective agency.

## Footnotes

1 Some informants wished to remain anonymous also with regard to their respective organizations.
